# MRI texture-based machine learning models for the evaluation of renal function on different segmentations: a proof-of-concept study

**DOI:** 10.1186/s13244-023-01370-4

**Published:** 2023-02-06

**Authors:** Xiaokai Mo, Wenbo Chen, Simin Chen, Zhuozhi Chen, Yuanshu Guo, Yulian Chen, Xuewei Wu, Lu Zhang, Qiuying Chen, Zhe Jin, Minmin Li, Luyan Chen, Jingjing You, Zhiyuan Xiong, Bin Zhang, Shuixing Zhang

**Affiliations:** 1 Department of Radiology, The First Affiliated Hospital of Jinan University, No. 613, Huangpu West Road, Tianhe District, Guangzhou, 510627 Guangdong People’s Republic of China; 2 Department of Radiology, Huizhou Municipal Central Hospital, No. 41 Eling Bei Road, Huizhou, 516001 Guangdong People’s Republic of China

**Keywords:** Chronic renal insufficiency, Glomerular filtration rate, Magnetic resonance imaging, Texture analysis, Machine learning

## Abstract

**Background:**

To develop and validate an MRI texture-based machine learning model for the noninvasive assessment of renal function.

**Methods:**

A retrospective study of 174 diabetic patients (training cohort, *n* = 123; validation cohort, *n* = 51) who underwent renal MRI scans was included. They were assigned to normal function (*n* = 71), mild or moderate impairment (*n* = 69), and severe impairment groups (*n* = 34) according to renal function. Four methods of kidney segmentation on T2-weighted images (T2WI) were compared, including regions of interest covering all coronal slices (All-K), the largest coronal slices (LC-K), and subregions of the largest coronal slices (TLCO-K and PIZZA-K). The speeded-up robust features (SURF) and support vector machine (SVM) algorithms were used for texture feature extraction and model construction, respectively. Receiver operating characteristic (ROC) curve analysis was used to evaluate the diagnostic performance of models.

**Results:**

The models based on LC-K and All-K achieved the nonsignificantly highest accuracy in the classification of renal function (all *p* values > 0.05). The optimal model yielded high performance in classifying the normal function, mild or moderate impairment, and severe impairment, with an area under the curve of 0.938 (95% confidence interval [CI] 0.935–0.940), 0.919 (95%CI 0.916–0.922), and 0.959 (95%CI 0.956–0.962) in the training cohorts, respectively, as well as 0.802 (95%CI 0.800–0.807), 0.852 (95%CI 0.846–0.857), and 0.863 (95%CI 0.857–0.887) in the validation cohorts, respectively.

**Conclusion:**

We developed and internally validated an MRI-based machine-learning model that can accurately evaluate renal function. Once externally validated, this model has the potential to facilitate the monitoring of patients with impaired renal function.

**Supplementary Information:**

The online version contains supplementary material available at 10.1186/s13244-023-01370-4.

## Key points

1. Texture analysis based on coronal T2-weighted MR images could evaluate the renal function in patients with diabetes.
2. The All-K and LC-K outperformed other segmentation methods in the evaluation of renal function impairment.
3. The segmentation methods could affect the results of renal function evaluation and the integrity of the coronal slices was crucial for renal imaging texture analysis.

**Supplementary Information:**

The online version contains supplementary material available at 10.1186/s13244-023-01370-4.

## Background

Chronic kidney disease (CKD) is a global health and economic burden and a raising cause of global deaths [1]. Diabetes and hypertension are the two main causes of CKD and subsequent end-stage kidney disease [2]. Early detection of renal function impairment is of great importance for promptly potent treatment strategies and eventually preventing renal dysfunction deterioration [3]. Chronic hypoxia and fibrosis are a vicious circle leading to renal functional and structural damages [4]. In clinical settings, it is meaningful to develop accurate and reliable biomarkers for detecting early renal function impairment and monitoring the disease progression.

Functional magnetic resonance imaging (fMRI) has been used to evaluate renal oxygenation and fibrosis [5–7]. Blood oxygenation level-dependent (BOLD) and arterial spin labeling (ASL) MRI has been performed to assess renal oxygenation changes as the renal function declined [8, 9]. Diffusion tensor imaging (DTI) and intravoxel incoherent motion diffusion-weighted imaging (IVIM-DWI) are semiquantitative methods to assess the fibrotic process during the chronic renal dysfunction [10, 11]. The fMRI techniques allow for quantifying and visualizing the renal function impairment on images; however, they suffer from low interobserver reproducibility and additional time and cost. Thus, fMRI is still not routinely used to evaluate the renal function.

With the advances in image analysis techniques in recent years, we can extract invisible textural features from MR images, which provides meaningful information to reflect the tissue heterogeneity [12, 13]. The degree of fibrosis was correlated with the imaging textures extracted from computed tomography and MRI on liver cirrhosis [14, 15]. Similarly, imaging textures of kidneys are potential biomarkers to reflect fibrosis and determine the estimated glomerular filtration rate (eGFR) and CKD status [16]. On MRI, the abundant features were indicative of renal function changes. The T2-weighted images (T2WI) are a commonly used sequence that can demonstrate the structures, cellular edema, fibrils and other underlying pathophysiology of some diseases [17, 18]. Texture analyses on T2WI have been used to evaluate the early renal function of the transplanted kidney and diabetic nephropathy, respectively [18, 19]. Therefore, the texture analysis on T2WI is able to reflect the pathological status of kidneys. However, the ROIs were variable in previous renal studies, which affect the process of model construction and performance [16, 18–21]. Thus, we aimed to investigate the value of texture analysis on T2WI in evaluating renal function and standardize the renal ROI segmentation method.

## Methods

### Subjects

This observational cohort study was approved by the institutional review board of our hospital. Written informed consent was obtained from each participant for renal MRI. Consecutive patients with hypertension and diabetes between October 2013 and November 2019 were recruited prospectively for renal MRI examination, and as the technique of imaging processing method developed, we retrospectively reviewed and secondary analyzed their images. The serum creatinine was obtained before and after one week of the date of MRI examination and eGFR was calculated by CKD Epidemiology Collaboration (CKD-EPI) 2009 (formula in Additional file 1). Patients with CKD1 (eGFR ≥ 90 mL/min/1.73 m^2^) are regarded as normal renal function, CKD2 and CKD3 (30 mL/min/1.73 m^2^ ≥ eGFR < 90 mL/min/1.73 m^2^) as mild or moderate renal function impairment, and CKD4 and CKD5 (eGFR < 30 mL/min/1.73 m^2^) as severe renal function impairment. The exclusion criteria were as follows: (1) coronal T2WI with severe artifacts; (2) patients with polycystic kidney diseases or acute renal injury; (3) patients who were using medications that can affect the creatinine level (e.g., cimetidine, trimethoprim, or cefotaxime); and (4) patients with poor control of hypertension. Finally, a total of 174 patients were enrolled for analysis. The patients were randomly assigned to the training cohort (*n* = 123) and the validation cohort (*n* = 51).

### MRI acquisition and preprocessing

All patients underwent MR examination at 3.0 T (Discovery MR 750, GE Healthcare, Milwaukee, WI, USA). The acquisition parameters were as follows: axial T1WI: repetition time (TR) = 6.2 (ms), echo time (TE) = 2.8 (ms), field of view (FOV) = 100 (mm^2^), matrix = 512 × 512, slice thickness = 4 mm, flip angle = 20°, bandwidth = 162.773 Hz/pixel; Coronal T2WI: TR = 1984 (ms), TE = 70 (ms), FOV = 100 (mm^2^), matrix = 512 × 512, slice thickness = 4.0 mm, flip angle = 90°, bandwidth = 325.508 Hz/pixel. Due to the differences between patients, the intensity range of T2WI was normalized to 0–255 by mean–variance normalization to reduce the influence on texture analysis.

### MR kidney image segmentation

**Fig. 1:**
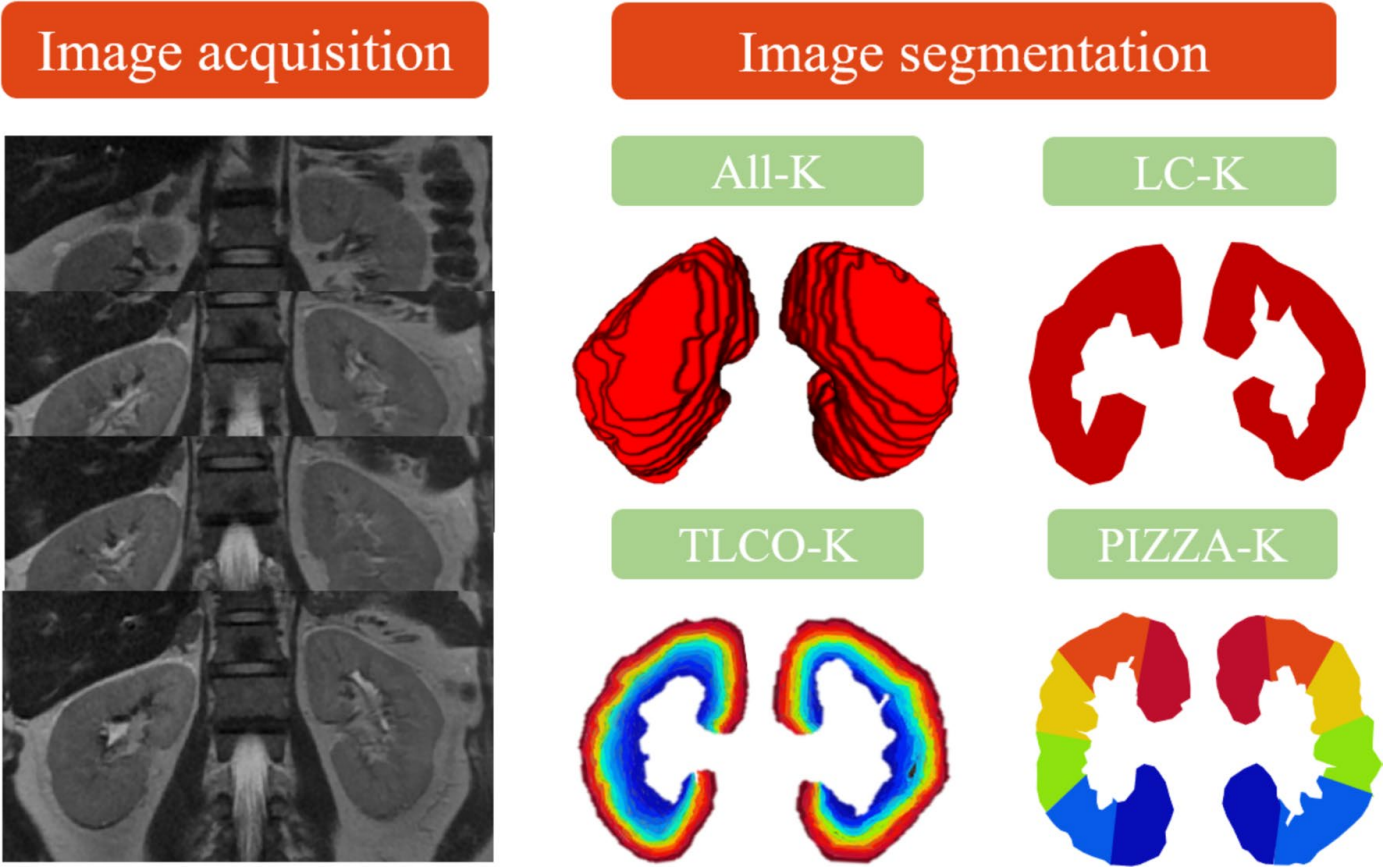
The four segmentation methods on the model construction for renal function evaluation. *Note:* All-K, all coronal slices of kidneys; LC-K, the largest coronal slices of kidneys; TLCO-K, 12 layers of the largest coronal slices of kidneys; PIZZA, 6 pieces of the largest coronal slices of kidneys

The bilateral kidneys were manually segmented on coronal T2WI using ITK-SNAP software (version 3.6.0, https://itk.org/) by a radiologist with 5-year diagnostic experience (Z.C., reader 1). Then 30 patients were randomly selected and segmented by a radiologist with 8-year diagnostic experience (X.M., reader 2) and reader 1 again. Disagreements between 2 readers reached a consensus through a discussion with an expert on abdominal radiology. To assess the influence of segmentation methods on the results of models for renal function evaluation, we compared four different kidney segmentation methods: (1) All-K: all coronal slices of renal parenchyma were delineated on two kidneys; (2) LC-K: the largest coronal slices crossing renal hilum of bilateral renal parenchyma were outlined; (3) TLCO-K: the largest coronal slices were segmented concentrically from the outer to the inner layers into 12 equal layers; (4) PIZZA-K: the largest coronal slices were segmented like a pizza, renal hilum as the center of the circle, upper and lower poles as edges, and it was divided into 6 parts with the equal degree. The four types of segmentation are demonstrated in Fig. 1.

### Texture extraction and model construction

The speeded up robust features (SURF) algorithm was used to detect and describe the local imaging features. Corner points were identified and 64 descriptor vectors were generated to describe each interest point. Because the corners points conducted by SURF from each ROI were different, the number of descriptor vectors was thus different. The process of SURF is described in Additional file 1. To obtain the same number of imaging features in the four segmentation methods, the bag of words (BoW) model was used to cluster similar features and describe their frequencies. The reduced imaging features by BoW were used for features selection and model construction. The reproducibility of features was evaluated by inter-/intraclass correlation coefficients (ICCs). When ICCs were more than 0.75, features were with good reproducibility. Additionally, age and gender were incorporated for their contribution to calculating eGFR. Both the imaging features and clinical characteristics entered into the support vector machine (SVM) to build a T2WI-based model for renal function evaluation. The receiver operating characteristic (ROC) curve analysis was used to evaluate the performance of SVM models, including area under the curve (AUC), sensitivity, specificity, and accuracy. We developed the models in the training cohort and validated them in the validation cohort.

### Statistics

An independent samples t-test and χ^2^ test were used to compare the clinical characteristics of training and validation cohorts when appropriate. Sensitivity, specificity, accuracy, and AUC were used to evaluate the performance of models. Delong test was used to compare AUC values. *p* < 0.05 with two-sided was considered significant. All statistical analyses were implemented in 2018 Python 3.6.5 (https://www.python.org/). “NumPy” package was used to standardize the intensity range of T2WI. “OpenCV” was applied for image masking, kidney segmentation, and feature extraction. “random” package was used for random assignment of training and validation cohorts. “pengouin” package was used to calculate ICCs. “scikit-learn” package was used for SVM. The ROC curves were plotted by the “matplotlib” package.

## Results

In a total of 174 patients were analyzed among 188 patients, 9 of them were excluded for motion artifacts on T2WI, and 5 of them were excluded for multiple renal cysts (occupying one-third of the renal parenchyma). Among 174 patients, 71 were categorized into the normal renal function group, 69 into the mild-moderate renal function impairment group, and 34 into the severe renal function impairment group. They were randomly assigned to the training cohort and the validation cohort (Table 1). No significant difference existed between training and validation cohorts (*p* > 0.05).

**Table 1:** The clinical characteristics of training and validation cohorts

Characteristics	Training cohort (*n* = 123)	Validation cohort (*n* = 51)	*p*
Age (years, mean)	56	56	0.521
Gender (%)			0.104
Male	79 (64%)	26 (51%)	
Female	44 (36%	25 (49%)	
Groups (%)			0.878
Normal renal function	50 (41%)	21 (41%)	
Mild-moderate renal function impairment	50 (41%)	19 (37%)	
Severe renal function impairment	23 (18%)	11 (22%)	

No significant difference of clinical characteristics was existed between training and validation cohorts

### Reproducibility of features among four segmentations

Bilateral kidneys were outlined by reader 1 and reader 2 and ICCs were analyzed. More than 83% of features in All-K, LC-K, TLCO-K, and PIZZA-K segmentations were reproducible (ICCs > 0.75).

### Performance of the four models in identifying normal renal function

**Fig. 2:**
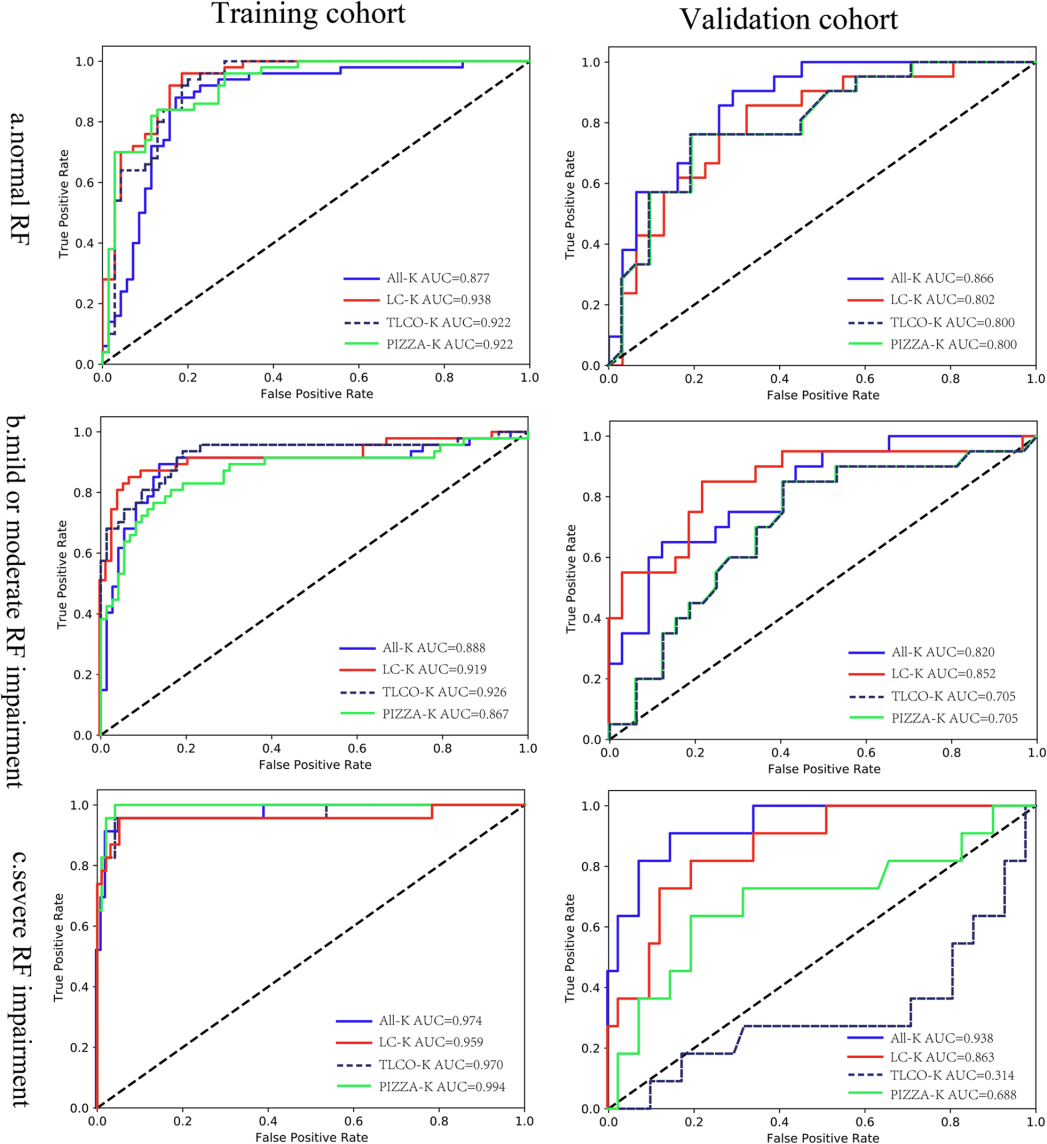
The ROC curves of four models on normal renal function, mild or moderate and severe renal function impairment in the training and validation cohorts. *Note:* ROC, receiver operating characteristic; RF, renal function

The four models based on different segmentation methods showed good performance in identifying the patients with normal renal function (Table 2 and Fig. 2a). In the training cohort, the All-K-, LC-K-, TLCO-K-, and PIZZA-K-based models yielded an AUC of 0.877 (95%CI 0.870–0.884), 0.938 (95%CI 0.935–0.940), 0.922 (95%CI 0.912–0.928), and 0.922 (95%CI 0.914–0.929), respectively, while in the validation cohort, the four models yielded an AUC of 0.866 (95%CI 0.863–0.870), 0.802 (95%CI 0.800–0.807), 0.800 (95%CI 0.785–0.816), and 0.800 (95%CI 0.784–0.958), respectively. No significant difference was found among the four models in identifying the normal renal function (All *p* values > 0.05).

**Table 2:** Diagnostic performance of four models in the evaluation of normal renal function

	Training cohort				Validation cohort			
Models	AUC	Sensitivity (%)	Specificity (%)	Accuracy (%)	AUC	Sensitivity (%)	Specificity (%)	Accuracy (%)
All-K	0.877 (0.870–0.884)	74.0 (73.5–74.5)	90.0 (89.4–90.6)	83.3 (82.8–83.9)	0.866 (0.863–0.870)	76.2 (75.0–78.2)	74.2 (71.3–74.5)	75.0 (74.5–75.5)
LC-K	0.938 (0.935–0.940)	84.0 (82.9–85.1)	91.4 (90.1–92.7)	88.3 (87.5–89.2)	0.802 (0.800–0.807)	79.2 (75.6–76.8)	83.9 (82.3–85.5)	80.8 (80.2–81.4)
TLCO-K	0.922 (0.912–0.928)	82.0 (81.4–82.6)	84.3 (82.4–86.2)	83.3 (82.3–83.9)	0.800 (0.785–0.816)	57.1 (55.7–58.5)	80.6 (79.9–81.3)	71.2 (70.6–71.7)
PIZZA-K	0.922 (0.914–0.929)	72.0 (71.5–72.5)	90.0 (89.9–90.0)	82.5 (81.7–83.3)	0.800 (0.784–0.958)	85.7 (85.1–86.2)	51.6 (51.1–52.1)	65.4 (64.9–65.9)

Data in parentheses are 95% confidence interval

### Performance of the four models in identifying mild or moderate renal function impairment

The four models based on different segmentation methods showed good performance in identifying the patients with mild or moderate renal function impairment (Table 3 and Fig. 2b). In the training cohort, the All-K-, LC-K-, TLCO-K-, and PIZZA-K-based models achieved an AUC of 0.888 (95%CI 0.881–0.895), 0.919 (95%CI 0.916–0.922), 0.926 (95%CI 0.919–0.932), and 0.867 (95%CI 0.860–0.874), respectively, while in the validation cohort, the four models achieved an AUC of 0.820 (95%CI 0.817–0.823), 0.852 (95%CI 0.846–0.857), 0.705 (95%CI 0.691–0.720), and 0.705 (95%CI 0.690–0.721), respectively. No significant difference was observed among the four models in identifying the mild or moderate renal function impairment (All *p* values > 0.05).

**Table 3:** Diagnostic performance of four models in the evaluation of mild or moderate renal function impairment

	Training cohort				Validation cohort			
Models	AUC	Sensitivity (%)	Specificity (%)	Accuracy (%)	AUC	Sensitivity (%)	Specificity (%)	Accuracy (%)
All-K	0.888 (0.881–0.895)	78.9 (78.2–79.2)	89.0 (88.4–89.6)	85.0 (84.4–85.6)	0.820 (0.817–0.823)	65.0 (63.8–66.2)	74.2 (73.9–74.5)	75.0 (74.5–75.5)
LC-K	0.919 (0.916–0.922)	83.0 (81.8–84.1)	94.5 (93.2–95.8)	88.3 (87.5–89.2)	0.852 (0.846–0.857)	65.0 (64.4–65.6)	84.4 (82.8–86.0)	76.9 (76.3–77.5)
TLCO-K	0.926 (0.919–0.932)	80.8 (80.3–81.4)	91.8 (89.9–93.6)	87.5 (86.9–88.1)	0.705 (0.691–0.720)	75.0 (73.6–76.3)	59.4 (58.7–60.0)	65.4 (64.8–66.0)
PIZZA-K	0.867 (0.860–0.874)	63.8 (63.3–64.3)	93.1 (93.1–93.2)	81.7 (80.9–82.4)	0.705 (0.690–0.721)	25.0 (24.4–25.5)	87.5 (87.0–87.9)	63.5 (62.9–64.0)

Data in parentheses are 95% confidence interval

### Performance of the four models in identifying severe renal function impairment

The All-K- and LC-K-based models showed excellent performance in identifying severe renal function impairment (Table 4 and Fig. 2c). The All-K-based model obtained an AUC of 0.974 (95%CI 0.967–0.982) and 0.938 (95%CI 0.934–0.941) in the training and validation cohorts, respectively. The LC-K-based model demonstrated an AUC of 0.959 (95%CI 0.956–0.962) and 0.863 (95%CI 0.857–0.887) in the training and validation cohorts, respectively. However, the performance of TLCO-K- and PIZZA-K-based models showed a big gap in the two cohorts, suggesting potential model overfitting. The TLCO-K-based model obtained an AUC of 0.970 (95%CI 0.964–0.977) in the training cohort whereas 0.314 (95%CI 0.201–0. 426) in the validation cohort. The PIZZA-K-based model obtained an AUC of 0.994 (95%CI 0.986–1.00) in the training cohort and 0.688 (95%CI 0.673–0.704) in the validation cohort. The All-K-based model showed significantly better performance than TLCO-K- and PIZZA-K-based models in the validation cohort (*p* = 0.009 and 0.009, respectively).

**Table 4:** Diagnostic performance of four models in the evaluation of severe renal function impairment

	Training cohort				Validation cohort			
Models	AUC	Sensitivity (%)	Specificity (%)	Accuracy (%)	AUC	Sensitivity (%)	Specificity (%)	Accuracy (%)
All-K	0.974 (0.967–0.982)	78.3 (77.8–78.7)	86.6 (86.0–87.2)	85.0 (84.4–85.6)	0.938 (0.934–0.941)	54.5 (53.5–55.6)	95.1 (94.7–95.6)	73.1 (72.5–73.6)
LC-K	0.959 (0.956–0.962)	87.0 (85.8–88.1)	90.7 (89.5–92.0)	90.0 (89.2–90.8)	0.863 (0.857–0.887)	72.7 (72.1–73.4)	87.8 (86.2–89.4)	84.6 (84.0–85.3)
TLCO-K	0.970 (0.964–0.977)	73.9 (73.4–74.4)	92.8 (90.9–94.7)	89.2 (88.5–89.8)	0.314 (0.201–0.426)	0 (-0.2–0.2)	85.3 (84.4–86.2)	67.3 (66.7–67.9)
PIZZA-K	0.994 (0.986–1.00)	82.6 (81.9–83.3)	76.2 (76.1–76.5)	77.5 (76.8–78.2)	0.688 (0.673–0.704)	9.1 (8.8–9.4)	78.0 (77.5–78.6)	80.6 (79.8–81.3)

Data in parentheses are 95% confidence interval

## Discussion

This current study demonstrated the good performance of texture-based MRI models in the evaluation of renal function impairment. The four machine learning models were similar in identifying normal renal function (AUC ≥ 0.800 in the validation cohorts). Interestingly, All-K- and LC-K-based models were more stable and reliable than TLCO-K- and PIZZA-K-based models when assessing impaired renal function.

T2WI is a routine and stable sequence and decreased signal intensity represents more fibrocollagenous stroma within the tissue. As fibrosis is the result of renal ischemia and inflammation, the degree of fibrosis reflects the status of renal function [22]. Subtle renal fibrosis is not identifiable with naked eyes based on T2WI, but the length of kidneys decreases as renal function deteriorates [23]. These subtle changes are transferred into the changes of a great number of texture features. Texture analysis on T2WI has been investigated in classifying the stages of liver cirrhosis [24]. But as for hemosiderin sedation and fibrosis in liver cirrhosis, hypointense hemosiderin on T2WI masks the fibrotic changes and lead to poor performance in further research [25]. However, renal fibrosis is mainly characterized by glomerulosclerosis, tubulointerstitial fibrosis, and tubular atrophy but a certain of hemosiderin deposition. Therefore, renal fibrosis results in more significant change of signal intensity on T2WI than hemosiderin deposition. On the other hand, the DWI, DTI, and IVIM were generated by changing scanning parameters based on T2WI, which has made the underlying fibrosis visible and measurable. Therefore, renal texture analysis on T2WI has provided an alternative method for evaluating the degree of renal fibrosis and detecting early renal function impairment.

ROI segmentation is an essential step in texture analysis and subsequent model construction. The models based on 4 segmentations have achieved good performance in training and validation cohorts when identifying normal renal function. And it has revealed that normal renal structure changed too little to yield difference among the whole kidneys, the largest coronal slices, or subregional segmentations of the largest coronal slices on texture analysis. However, subtle structures changed as renal function declined. The All-K- and LC-K-based models outperformed the TLCO-K- and PIZZA-K-based models in assessing the mild or moderate and severe renal function impairment. It may infer that the integrity of coronal slices is significant to reflect the renal texture changes during the chronic process of renal dysfunction. Meanwhile, the All-K consisted of LC-K and other coronal slices, but the All-K-based model did not significantly better than the LC-K-based model. This may be due to feature redundancy from too many coronal ROI sections in All-K. Feature redundancy referred to the strong correlations between texture features among slices. Thus, the imaging textures from LC-K were representative of the corresponding kidney. Our finding was in accordance with previous fMRI studies which measured the values on the coronal slices [26, 27]. Both All-K and LC-K were feasible segmentation methods for establishing a robust and effective model to evaluate renal function.

Renal function was determined by the glomerular mainly in the cortex and renal tubules in the medulla. The remarkable pathological changes of advanced renal dysfunction blurred the boundary between the renal cortex and medulla. The cortico-medullary areas were related to renal oxygenation, eGFR and the progression of chronic kidney diseases [28, 29], yet their textures were under-investigated in TLCO-K and PIZZA-K segmentations. The performance of the PIZZA-K-based model was better than the TLCO-K-based model in identifying severe renal function impairment. It might reveal that the integrity of cortico-medullary areas on texture analysis was important in evaluating renal function. Meanwhile, the abundant texture features from layers or pieces led to overfitting in the training cohort when identifying the severe renal function impairment. The overfitting suggested the TLCO-K- and PIZZA-K-based models were rather complicated and their generalization ability was poor. The indifferent results referred that the separation of texture analysis of renal cortex or medulla or segmental renal areas was inappropriate in the evaluation of impaired renal function. Although the imaging models built by the subregional ROI analysis within the lesion outperformed the ones from the primary whole lesion on tumor studies for the abundant heterogeneity [30–32], the subregional ROI texture analysis may not show good performance in non-tumoral diseases. Our study has proven that texture analysis models based on the integral largest coronal slices or the whole organs for chronic kidney diseases showed a satisfying performance.

This study has some limitations. First, the sample size was relatively small. MRI examination is not a routine examination for patients with chronic renal function impairment. We categorized patients into 3 renal function groups on evaluation of MRI-texture-based models for the uneven distribution of eGFR. Second, we only investigated the four ROI segmentations on coronal slices of T2WI. The predictive value of various segmentations on axial slices remains unknown, which needs further study. However, the largest coronal slices contained more information than the largest axial slices, and the former has been commonly used in fMRI studies. Thirdly, our study was based on T2WI only, the predictive value of other MRI or fMRI sequences was not discussed. T2WI as a routine sequence makes it more convenient to obtain. Moreover, the models constructed from T2WI were easy-to-use in other institutions compared to the extra fMRI.

In conclusion, we developed and validated a texture-based MRI model to evaluate renal function, which offers an alternative tool to detect renal impairment. Furthermore, texture analysis on MRI should be on the integral coronal slices or the whole coronal slices of the kidney, which would develop more robust models than the subregional segmentations-based models for assessing the different status of renal function. The stable segmentations on renal MRI texture analysis will promote future studies of other nephropathies and predict the risk of renal function decline of CKD.

## Supplementary Information

**Additional file 1.** Supplementary materials.

## Data Availability

The datasets generated and/or analyzed during the current study are not publicly available due to the ongoing further studies but are available from the corresponding author on reasonable request.

## Abbreviations

All-K: All coronal slices
ASL: Arterial spin labeling
BOLD: Blood oxygenation level-dependent
CKD: Chronic kidney diseases
DTI: Diffusion tensor imaging
DWI: Diffusion-weighted imaging
eGFR: Estimated glomerular filtration rate
IVIM: Intravoxel incoherent motion
LC-K: The largest coronal slice
PIZZA-K: Six pieces segmentation of the largest coronal slice
ROI: Region of interest
SURF: Speeded up robust features
T2WI: T2-weighted images
TLCO-K: Twelve concentric objective segmentation of the largest coronal slice
