# Supplementary material for: MRI texture-based machine learning models for the evaluation of renal function on different segmentations: a proof-of-concept study
Source: Insights Imaging. 2023 Feb 6;14:28. doi: 10.1186/s13244-023-01370-4 (PMC9902579; doi:10.1186/s13244-023-01370-4)
Supplement: Supplementary file 1 — Additional file 1. Supplementary materials. [file 13244_2023_1370_MOESM1_ESM.pdf]

ELECTRONIC SUPPLEMENTARY MATERIAL

MRI texture-based machine learning models for the evaluation of renal function on different segmentations: a proof-of-concept study

1. The CKD Epidemiology Collaboration (CKD-EPI) 2009 is as followed:

$$eGFR = 141 \times \min\left(\frac{Scr}{k}, 1\right)^{\alpha} \times \max\left(\frac{Scr}{k}, 1\right)^{-1.209} \times 0.993^{Age} \times 1.018(if\ female) \times 1.159(if\ black)$$

k = 0.7 if female

k = 0.9 if male

α = -0.329 if female

α = -0.411if male

Scr = serum creatinine (mg/dl)

2. The steps to detect and describe the imaging features.

The Speeded up robust features (SURF) algorithm was used to detect and describe the local imaging features with the following steps. Hessian matrix was generated to localize the image points, and box filters were used to approximate the second-order Gaussian derivatives of each image point to reduce the calculation time. The interest points were obtained when they achieved the maximum value among the surrounding voxels. Then, the circular region around each interest point was orientated. The Harr wavelets in horizontal and vertical directions were dx and dy. Sum up the dx and dy of the point of interest in the 60-degree sector in the circle. The sector is rotated at an interval of 0.2 radians and Harr wavelets were calculated again. The main direction is determined by the maximum Harr wavelet value. Then, a 4×4 square region is

constructed along with the selected main orientation, the Harr wavelets were calculated in each subregion and 64 SURF descriptors are extracted.
